# Dr. Thomas Watson

**Published:** 1859-03

**Authors:** 


DR. THOMAS WATSON.—We are happy to announce the appointment of the distinguished author of the well-known "Lectures on the Principles and Practice of Physic," as Physician Extraordinary to the Queen, in place of Dr. Bright, who has so recently died. This appointment has given universal satisfaction to the profession of England.

